# The role of tea in managing cardiovascular risk factors: potential benefits, mechanisms, and interventional strategies

**DOI:** 10.3389/fnut.2025.1530012

**Published:** 2025-04-24

**Authors:** Ziming Xu, Zhu Tao, Yan Guo

**Affiliations:** ^1^Xiyuan Hospital Affiliated to China Academy of Chinese Medical Sciences, Beijing, China; ^2^The Chinese Journal of Integrated Traditional and Western Medicine Press, Beijing, China; ^3^WHO Collaborating Center for Traditional Medicine, Institute of Chinese Materia Medica, China Academy of Chinese Medical Sciences, Beijing, China

**Keywords:** tea, cardiovascular risk factors, non-pharmacological treatment, nutritional intervention, interventional strategies

## Abstract

Traditional major cardiovascular disease (CVD) risk factors include dyslipidemia, hypertension, smoking, diabetes, and obesity. Tea is rich in various bioactive substances such as tea polyphenols, theaflavins, and tea polysaccharides. Due to the regulatory effects on multiple pathways and its anti-inflammatory and antioxidant properties, these active substances have shown significant efficacy in regulating dyslipidemia, hypertension, diabetes, obesity, and cardiac autonomic function. Additionally, tea possesses anti-inflammatory and antithrombotic properties, making it a promising dietary supplement for nutritional interventions in the primary and secondary prevention of CVDs. However, the complex composition of tea, although shown to have certain effects *in vivo*, does not fully elucidate the specific mechanisms of action. Moreover, the varying application methods across different studies lead to differences in intervention effects and dose–response relationships, sometimes resulting in contradictory findings. This article reviews the potential benefits, mechanisms of action, and application methods of tea for cardiovascular risk factors, elucidating its potential as a nutritional intervention.

## 1 Introduction

Cardiovascular diseases (CVDs) are significantly influenced by several risk factors, including dyslipidemia, hypertension, smoking, diabetes mellitus, and obesity (1). These factors are widely recognized not only in adult populations but are increasingly prevalent among children and adolescents. This shift has resulted in a higher incidence of early atherosclerosis and cardiovascular events (2, 3).

Obesity, in particular, exhibits a close relationship with other cardiovascular risk factors such as hypertension, diabetes, and hyperlipidemia, thereby forming a complex and interconnected network of risk factors (4). Additionally, smoking is a critical behavioral risk factor that further elevates the risk of CVDs, especially in individuals with other metabolic abnormalities (5).

Therefore, the early identification and management of these risk factors are essential to mitigate the incidence and mortality associated with cardiovascular diseases (6). Recent research has also identified additional potential independent risk factors, including gender differences, aging, and psychosocial factors, which warrant further exploration and consideration (7, 8).

The management of these risk factors includes both pharmacological and non-pharmacological approaches. Pharmacological treatments encompass lipid regulation, blood pressure control, diabetes treatment, as well as symptomatic treatment for other independent risk factors. For patients already diagnosed with atherosclerotic cardiovascular diseases, anti-inflammatory and antithrombotic interventions are also considered crucial for controlling underlying risk factors.

Non-pharmacological treatments include lifestyle modifications, nutritional interventions, physical activity, and psychological interventions. The importance of non-pharmacological treatments has been increasing in light of changes in social environment, shifts in health awareness, and the continuous development of adjunctive therapies. Numerous studies have confirmed that effective management of these risk factors can significantly improve disease symptoms and reduce the recurrence and rehospitalization rates of CVD, thereby extending patient life expectancy and improving quality of life.

Nutritional interventions cover four main areas: dietary structure, obesity and weight management, fat and sugar intake, and the use of dietary supplements. The goal is to prevent and actively improve cardiovascular diseases through nutritional diets. Tea, as a soft drink, is rich in phenols, polysaccharides, and flavonoids, and has high nutritional and health value. These components have been shown to have positive effects on reducing the risk and progression of CVD, making tea a suitable dietary supplement for cardiovascular disease needs (9). Tea can be classified into six categories based on the degree of fermentation: green tea, white tea, yellow tea, oolong tea, black tea, and dark tea. A large body of clinical and mechanistic research supports that the chemical components in tea have positive effects on lipids, blood pressure, diabetes, obesity, inflammation, and thrombosis.

## 2 Chemical composition of tea

Tea contains various chemical constituents including tea polyphenols, tea pigments, tea polysaccharides, tea saponins, alkaloids, aromatic substances, vitamins, amino acids, and a small amount of inorganic substances. Tea polyphenols and tea polysaccharides are considered the primary sources of the health benefits of tea, and their content varies depending on the type and degree of fermentation.

Catechins are the most significant chemical constituents of tea polyphenols, accounting for about 60–80% of the total tea polyphenols. They can be divided into four categories: epigallocatechin gallate (EGCG), epigallocatechin (EGC), epicatechin gallate (ECG), and epicatechin (EC) (10). During tea fermentation, some catechins are oxidized to theaflavins, resulting in a decrease in the total catechin content (from approximately 172.8 mg/g to 48.2 mg/g), and an increase in theaflavins (from approximately 17.9 mg/g to 43.4 mg/g) (11). The antioxidant activity of theaflavins is lower than that of catechins and includes four isomers: theaflavin (TF1), theaflavin-3-gallate (TF2A), theaflavin-3′-gallate (TF2B), and theaflavin-3,3′-digallate (TF3) (12).

**Figure 1:**
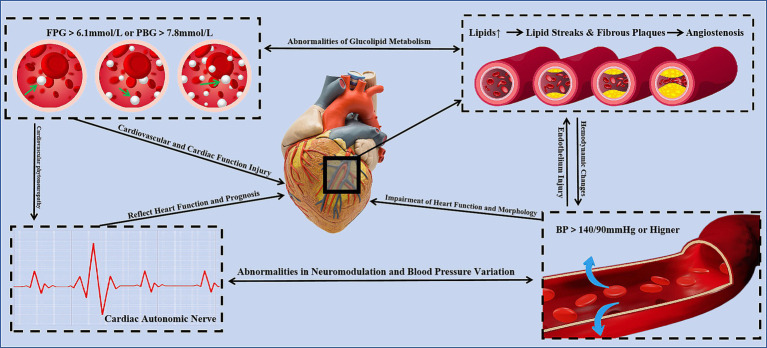
Heart and cardiovascular risk factors.

Tea polysaccharides are non-starch bound acidic polysaccharides composed of neutral sugars, uronic acids, and proteins. The monosaccharide components of tea polysaccharides primarily include rhamnose, arabinose, xylose, mannose, glucose, galactose, and fucose (13). The variation in the monosaccharide composition and content, as well as differences in polysaccharide structures across different types of tea, suggest that tea polysaccharides are unique components of tea (Figure 1).

## 3 Benefits and mechanisms of tea on cardiovascular risk factors

### 3.1 Lipids

Dyslipidemia is considered a critical factor in atherosclerosis and related cardiovascular events. The association between low-density lipoprotein and other apolipoprotein B-containing lipoproteins and cardiovascular event risk has also been confirmed through Mendelian randomization studies in humans (14). Moreover, studies have shown that tea consumption not only significantly improves hyperlipidemia but also reduces the risk of coronary atherosclerotic heart disease (15, 16).

Early studies attributed the lipid metabolism-enhancing effects of tea to the role of caffeine in promoting fat breakdown (17). However, recent findings demonstrate a significant positive correlation between the active components of tea and its lipid-lowering and antioxidant properties. These effects may be associated with increased excretion of bile acids and cholesterol, as well as enhanced activities of catalase and glutathione peroxidase (18). Cross-sectional studies indicate that women aged 20 to 48 derive higher average antioxidant capacity and anti-lipid oxidative damage benefits from dietary intake of total flavonoids and theaflavins in tea compared to vitamins and anthocyanins (19). Raederstorff et al. observed a reduction in intestinal cholesterol absorption rates in rats from 73.7 to 62.7% when exposed to an intervention of 0.5 g/kg epigallocatechin gallate (EGCG) compared to 0.1 g/kg. Correspondingly, *in vitro* experiments demonstrated that EGCG dose-dependently decreased cholesterol solubility in micelles, suggesting that it may improve lipid metabolism by inhibiting the solubilization of cholesterol in the digestive tract and reducing its absorption (20). Furthermore, thearubigins have been shown to elevate conjugated bile acid levels, suppress the intestinal FXR-FGF15 signaling pathway, reduce cholesterol and fat synthesis, and activate alternative bile acid synthesis pathways to promote fat breakdown (21). Kashif et al. examined the effects of catechins, theaflavins, and freeze-dried ginger extracts in hyperglycemic, obese, and liver-impaired rats. Their findings revealed that catechins significantly improved body weight, cholesterol (−11.03%), and low-density lipoprotein (LDL) (−14.25%), with enhanced results when combined with theaflavins (22). Fu et al. (131) further identified that theaflavin (TFDG) lowered fasting blood glucose and lipid concentrations by upregulating the Nrf2 signaling pathway and circ-ITCH expression. Their research validated the antioxidant properties of TFDG and its beneficial effects on liver and kidney function, as well as cellular structural integrity (23).

While research has shown that tea and its components can improve blood lipid levels, combining tea with other interventions may result in enhanced effects (24, 25). However, the relationship between tea-based intervention strategies and the regulation of blood lipids remains an area of significant interest. Elke et al. (132) conducted a study involving 102 participants with mild-to-moderate hypercholesterolemia (TC: 5.70 ± 0.74 and/or LDL-C: 3.97 ± 0.61 mmol/L) using tea components and cellulose as interventions. Although total cholesterol (TC) and low-density lipoprotein cholesterol (LDL-C) levels decreased over the intervention period, no statistically significant difference was observed compared to the placebo group. It was concluded that the lipid-lowering effects of tea could not be confirmed at a daily dosage of 75 mg theaflavins and 150 mg catechins, whether applied individually or in combination (26). Patricia et al. (133) found in their study on type 2 diabetes patients undergoing standard therapy and statin treatment that a 12-week intervention with 400 mg green tea extract (90% total polyphenols, 80% total catechins, 45% EGCG, and 1.0% caffeine) did not significantly alter blood lipid or glucose levels compared to placebo. However, the green tea extract positively impacted arterial health by reducing the central arterial augmentation index (−3.05 ± 10.8% vs. 6.7 ± 0.1%), suggesting an improvement in arterial stiffness (27). The dosages of tea components applied in the above studies showed no significant intervention effects; however, alternative dosages or the use of brewed tea were not further investigated. In contrast, Bianca et al. (134) evaluated a six-week intervention involving combined supplementation of fish oil (1.7 g EPA + DHA/day), plant sterol-enriched chocolate (2.2 g/day), and green tea (~170.8 mg/day) in 53 statin-intolerant type 2 diabetes patients. Responders (*n* = 10) continued with a 12-week statin dose-reduction combined with supplementation. No significant differences were observed in lipid levels or inflammatory response improvements compared to standard therapy (28). Shun et al. (135), in a double-blind, placebo-controlled study, demonstrated that a four-week intervention with 165 mg mono-glucosyl hesperidin and 387 mg green tea catechins significantly reduced triglyceride (TG) levels (29).

**Figure 2:**
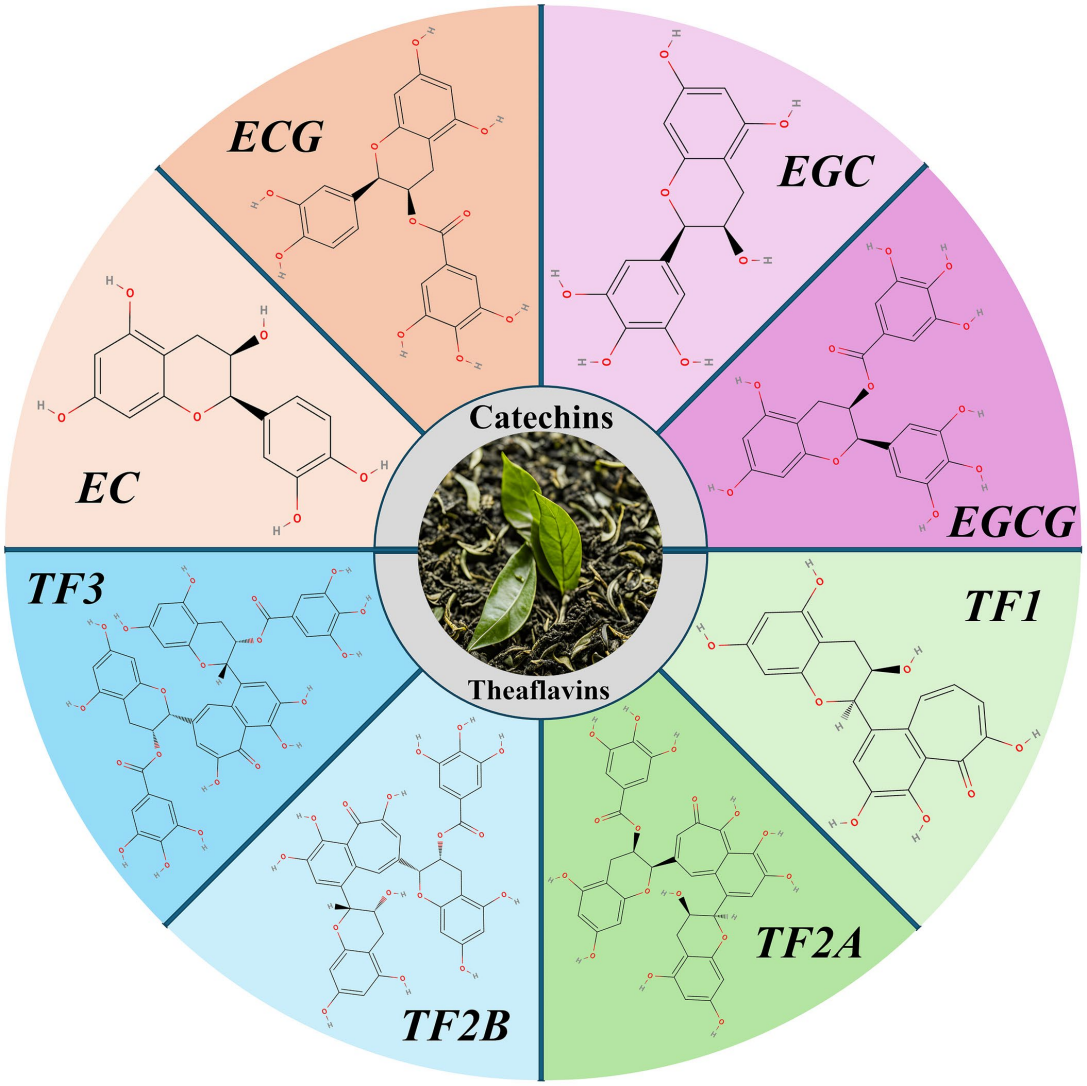
Chemical composition of tea.

In summary, tea’s lipid-improving effects may be more pronounced when combined with pharmaceutical or supplementary interventions, rather than relying solely on tea or its extracts. Independent application of tea extracts may not achieve optimal outcomes for blood lipid regulation (Figure 2).

### 3.2 Blood pressure

Hypertension is a common cardiovascular disease characterized by elevated blood pressure. It causes target organ damage through hemodynamic changes and, in conjunction with other risk factors, increases the risk of cardiovascular diseases and events (30). Retrospective studies by Jonathan et al. (136) observed that long-term regular tea consumption led to an average reduction of 2–3 mmHg in systolic and diastolic blood pressure in elderly women, with particularly notable effects at a daily intake of 250 mL. However, the study did not specify the actual concentration or type of tea consumed (31). Similarly, a study conducted in Taiwan involving 1,507 tea-drinking participants examined the impact of tea consumption on the risk of developing hypertension. The results revealed that habitual moderate tea consumption (120 mL/day for more than one year) significantly reduced the incidence of hypertension (32). Furthermore, a meta-analysis of randomized controlled trials demonstrated that tea components reduced oxidative stress and inflammatory responses while improving vasodilation associated with blood pressure changes (33). These findings provide supporting evidence for the long-term use of tea as a potential strategy for the prevention and management of hypertension.

**Figure 3:**
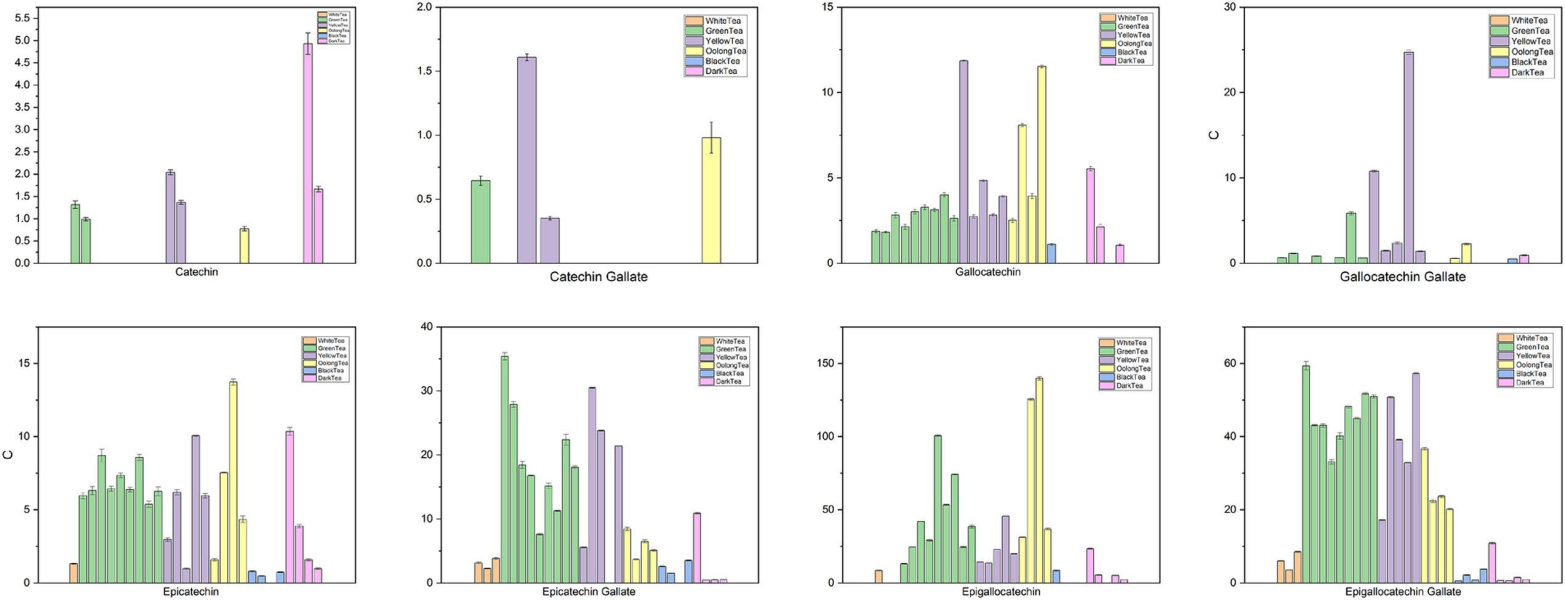
Chemical content of tea (93) (Sorted by white tea, green tea, yellow tea, oolong tea, black tea, and black tea categories. **White tea** includes Gongmei white tea, Shoumei white tea, White Peony tea; **green tea** includes Dianqing tea, Dongting Biluochun tea, Duyun Maojian tea, Enshi Yulu tea, Lu’an Guapian tea, Lushan Yunwu tea, Taiping Houkui tea, Xihu Longjing tea, Yongxi Huoqing tea; **yellow tea** includes Huoshan large yellow tea, Junshan Yinzhen tea, Mengding Huangya tea, Weishan Maojian tea, Yuan’an Luyuan tea; **oolong tea** includes Fenghuang Shuixian tea, Luohan Chenxiang tea, Tieguanyin tea, Wuyi Rock tea; **black tea** includes Dianhong Congou black tea, Keemun black tea, Lapsang Souchong black tea, Yichang Congou black tea; and **dark tea** includes Fuzhuan Brick tea, Liupao tea, Pu-erh tea, Qingzhuan Brick tea, Tibetan tea).

Furthermore, clinical research shows that acute tea consumption significantly increases systolic and diastolic blood pressure after 120 min, although subjects showed the lowest digital volume pulse stiffness index (34). Jonathan et al. (136) also confirmed that acute tea consumption raised systolic blood pressure within three hours (35). The acute rise in blood pressure from tea consumption may be due to caffeine, but long-term tea consumption has a positive effect on blood pressure regulation, potentially related to polyphenolic compounds (Figure 3).

Hyperhomocysteinemia-associated hypertension is gaining increasing attention in clinical diagnosis and treatment, as it is also a risk factor for atherosclerotic cardiovascular disease (36). Long-term tea consumption has been shown to improve endothelial function, reduce hemodynamic-mediated vascular dilation, and thereby improve blood pressure levels. These effects may be associated with the inhibition of angiotensin by tea pigments and theaflavins (37–39). Lili et al. (137) demonstrated that epigallocatechin gallate (EGCG), due to its potential antioxidant and anti-inflammatory activities, significantly improved cerebrovascular damage in rats induced by high Hcy levels. It also increased glutathione levels, enhancing antioxidant capacity (40). Tea polyphenols and bioflavonoids were found to inhibit DNMT-mediated DNA methylation, which partially restored HUVEC cell damage induced by high Hcy levels and reduced PAI-1 activity (41, 42). Notably, the risk of high Hcy levels may be positively correlated with the intake of black or green tea due to individual differences in the methylation of polyphenolic compounds. In contrast, oolong tea consumption was not associated with an increased risk of high Hcy levels. Moreover, theaflavins in oolong tea exhibited protective effects against Hcy-induced endothelial damage (43–45).

Hypertension and cellular damage are closely linked, and their treatment are crucial for improving cardiovascular diseases (46). Catechins, major antioxidant components of tea polyphenols, improve oxidative stress in cardiomyocytes by reducing reactive oxygen species production in cells and mitochondria and decreasing antioxidant factor consumption. This process may depend on the inhibition of inflammation-related pathways, leading to a reduction in interleukin-8 (IL-8) production (47). Aravind et al. (138) demonstrated that EGCG not only inhibited the NF-κB pathway, reducing the transcription of downstream inflammatory factor genes, but also decreased type II coronary endothelial cell extravasation and monocyte adhesion. This blocked cell activation and further validated the positive role of EGCG in mitigating inflammatory responses and maintaining vascular homeostasis (48).

The regulatory effects of tea on blood pressure vary across studies, which may be closely linked to differences in tea types and intervention dosages. Marjan et al. (139) conducted a meta-analysis of five RCT studies involving 408 participants, finding that habitual tea consumption reduced SBP and DBP by approximately −3.53 mmHg and − 0.99 mmHg, respectively. The blood pressure reduction was more pronounced with tea consumption durations of ≥3 months, with green tea showing greater efficacy in lowering blood pressure compared to black tea (49). Additionally, Biesinger et al. (140) used a combination of phytochemicals (grape seed and skin extract 330 mg, green tea 100 mg, resveratrol 60 mg, and a mixture of quercetin, ginkgo, and bilberry 60 mg) for placebo-controlled crossover intervention in metabolic syndrome subjects with elevated blood pressure, finding that phytochemical supplements reduced mean arterial pressure and diastolic pressure by 4.4 mmHg. Although participants’ blood pressure improved in this study, the complexity of the chemical combination used means the improvement cannot be solely attributed to green tea (50). A clinical randomized crossover trial found that taking three capsules of 500 mg green tea extract (260 mg polyphenols) daily for four weeks significantly reduced 24-h systolic blood pressure (−3.61 ± 1.23 vs. 1.05 ± 1.34 mmHg), daytime systolic blood pressure (−3.61 ± 1.26 vs. 0.80 ± 1.57 mmHg), and nighttime systolic blood pressure (−3.94 ± 1.70 vs. 1.90 ± 1.66 mmHg) in prehypertensive obese women, although there were no significant differences in diastolic pressure and other parameters (51).

### 3.3 Diabetes

**Figure 4:**
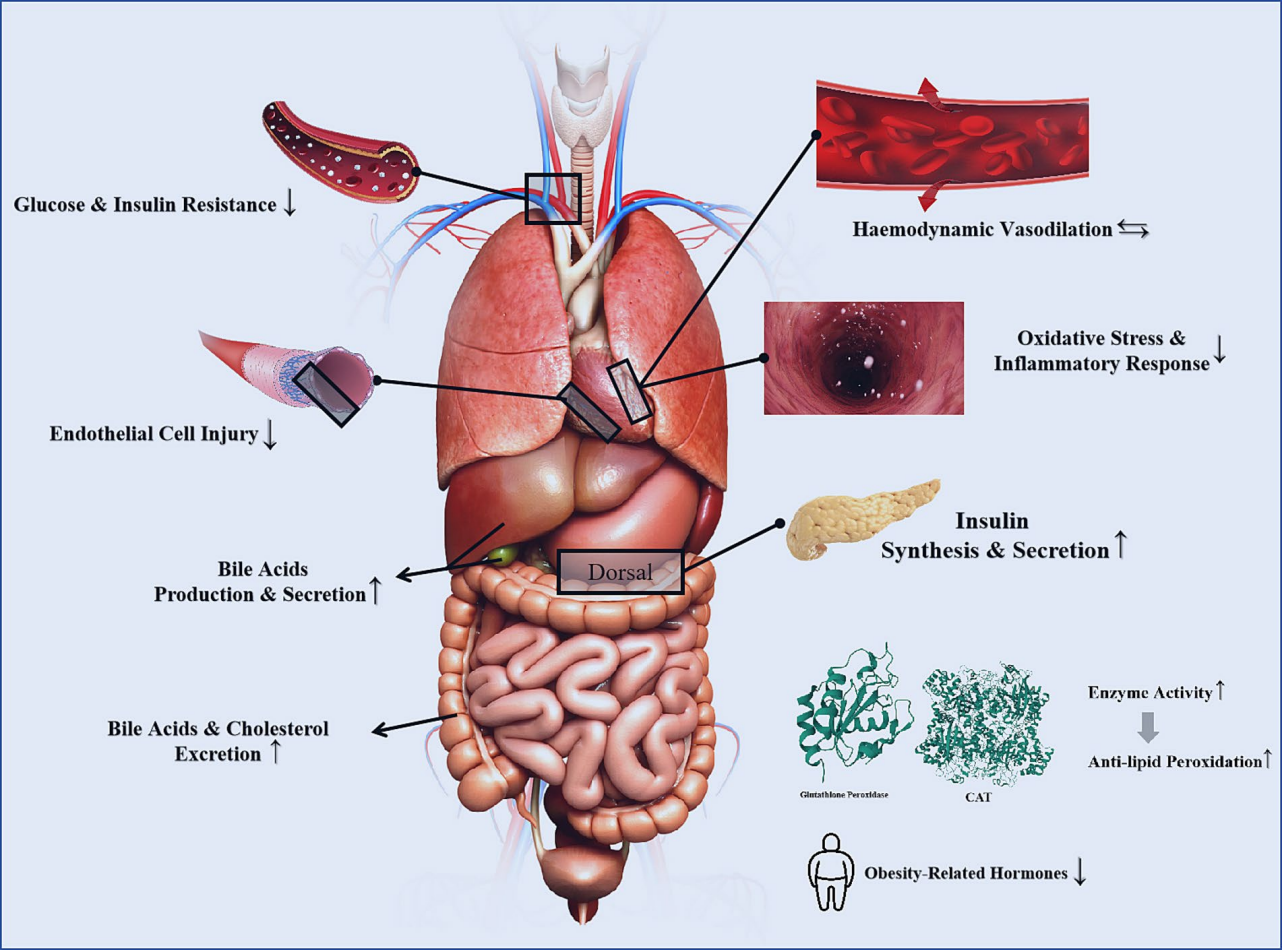
The mechanisms of tea for CVD risk factors [The 3D structures of Glutathione peroxidase and catalase are from RCSB PDB (94, 95)].

Type 1 diabetes, type 2 diabetes, and prediabetes are considered independent risk factors for CVDs. Patients with these conditions have a 2–4 times higher likelihood of developing CVDs compared to healthy individuals. When CVDs coexist, the risk of major cardiovascular events and all-cause mortality increases (52–55). Improving glycemic and lipid metabolism, controlling blood pressure, and reducing insulin resistance are critical strategies for mitigating the risk of diabetes as a CVD risk factor. A cohort study indicated that overweight/obese type 2 diabetes patients who consume more than 5 grams of green tea daily and have been drinking green tea for over 40 years are associated with a 50% reduction in cardiovascular disease risk (56). Mi et al. (141) discovered that the beneficial effects of EGCG on obesity and diabetes are attributed to its phosphorylation of AMPK and ACC, key enzymes that inhibit lipogenesis. Additionally, EGCG improves disruptions in redox balance and mitochondrial function, alleviating insulin signaling pathway blockages (57). Ren et al. (142), through an intervention in a high-fat diet and streptozotocin-induced type 2 diabetes (T2DM) mouse model, confirmed that EGCG reduced blood glucose levels and improved insulin resistance in T2DM mice. Moreover, EGCG regulated total cholesterol, triglyceride, and low-density lipoprotein receptor levels while reducing lipid deposition in vascular endothelial cells (58). Clearly, tea not only improves blood glucose levels and insulin resistance but also plays a positive role in regulating glucose-lipid metabolism and addressing obesity/overweight conditions (Figure 4).

Polysaccharides, catechins, and theaflavins in tea are considered primary contributors to its blood glucose-lowering effects, as they improve lipid synthesis, gut microbiota, and glucose transporter proteins (59–61). A randomized controlled trial targeting adults with metabolic syndrome found that the improvement in fasting blood glucose by green tea extract may be attributed to a reduction in blood endotoxins, suppression of intestinal inflammation, and decreased intestinal permeability (62). Yan et al. (143) observed that tea effectively inhibited weight gain, glucose metabolism disorders, and oxidative stress caused by a high-fat diet, while also improving gut microbiota composition (63). Green tea was shown to inhibit NF-κB pathway activation, which upregulated the expression of Glu2 and PPARγ, thereby reducing lipid synthesis and promoting glucose transport (64). Theaflavins, at non-cytotoxic doses, significantly enhanced glucose uptake in insulin-resistant cells. This activity was linked to the upregulation of GLUT4 expression and Akt phosphorylation. Moreover, theaflavins mitigated insulin resistance in hepatocytes induced by free fatty acids, indicating their potential role in improving metabolic abnormalities associated with insulin resistance (65).

Furthermore, Hadeel et al. (144) demonstrated that after three weeks of treatment with 100–200 mg/dL doses of green tea extract, wound-related parameters in the skin of T1DM rats significantly improved within 14 days. The intervention regulated the expression of microRNAs (miR-21, miR-23a, miR-146a, miR-29b) and apoptotic genes (Bax, caspase-3, BcL-2). These findings provide potential insights into the role of tea polyphenols in ameliorating apoptosis and promoting angiogenesis (66).

### 3.4 Obesity

Obesity is widely recognized as a significant risk factor for cardiovascular diseases (CVDs). Studies indicate a strong association between obesity and traditional cardiovascular risk factors such as hypertension, diabetes, and dyslipidemia, with obese individuals having markedly higher rates of these conditions compared to those of normal weight. Obesity negatively impacts key risk factor levels and mechanisms, increasing the risk of CVDs through mechanisms such as myocardial dysfunction, insulin resistance, inflammation, and abnormal metabolism and hormone secretion by adipose tissue (67–69).

A meta-analysis involving 3,802 participants revealed that green tea extract, as a supplementary intervention for cardiovascular diseases, effectively improved oxidative stress markers such as malondialdehyde and total antioxidant capacity, while also enhancing body composition and obesity-related hormones. Unfortunately, the dose–response evaluation did not yield optimal results, likely due to variations in the quality of evidence included in the analysis (70). *Joshua et al.* conducted a 12-week intervention among 55 overweight and/or obese adult participants using a multi-component supplement (50 mg forskolin, 500 mg green coffee bean extract, 500 mg green tea extract, 500 mg beetroot extract, 400 mg *α*-lipoic acid, 200 IU vitamin E, and 200 mg CoQ10) or a placebo. The trial group showed significant reductions in body weight and fat mass, along with improvements in obesity-related plasma markers (GDF15, miR-122, miR-34a), biomarkers for non-alcoholic fatty liver disease (AST, ALT), and resting energy metabolism (71).

Xie et al. (145), in a randomized controlled trial, investigated the effects of a 12-week intervention with decaffeinated green tea polyphenols (400 mg/day) or an equivalent placebo in obese girls aged 6–10. The decaffeinated green tea polyphenol group demonstrated significant reductions in body mass index, body fat percentage, waist circumference, and waist-to-hip ratio, with no adverse effects reported during the study period (72). Tea extracts may exert their effects by stimulating thermogenesis in brown adipose tissue through the interaction between catechin polyphenols and norepinephrine, which improves body weight and body fat levels (73). Additionally, the efficacy and safety of decaffeinated green tea extract in improving obesity and regulating sex hormone secretion in obese women have been clinically confirmed (74). Related animal studies have shown that the weight control effects of oolong tea extract are dose-dependent and unrelated to dietary changes, with the most significant weight changes observed at moderate doses (75).

### 3.5 Autonomic function

Cardiac autonomic function is closely related to coronary heart disease, atherosclerosis, and other circulatory system diseases. This may be due to the vascular spasms, platelet aggregation, and activation of inflammatory responses caused by dysregulation of sympathetic and parasympathetic nervous activities, which are common characteristics in the pathology of various heart diseases (76). Studies have confirmed that flavonoid-rich foods such as tea can inhibit excessive sympathetic activity, which may be one of the mechanisms through which these substances exert cardioprotective effects (77).

*In vivo* studies have demonstrated that black tea significantly increases the high-frequency power and the low-frequency/high-frequency power ratio in the electrocardiograms of rats, improving heart rate variability and enhancing the balance between vagus and sympathetic nerve activity (78). Michelle et al. (146) observed in hypertensive rat models that green tea intervention reduced renal sympathetic nerve activity, improved baroreceptor function, and significantly lowered blood pressure (79). *In vitro* research by Fujiko et al. (147) applied various catechin derivatives to the thoracic aortic endothelium of rats. These derivatives inhibited acetylcholine-induced endothelium-dependent relaxation to varying degrees, enhanced vascular reactivity, and exhibited neuromodulatory effects on vascular smooth muscle cells (80).

Heart rate variability (HRV) reflects cardiac autonomic nervous system activity and can quantitatively assess sympathetic and vagal tone, balance, and disease prognosis (81). Reduced HRV is associated with poor cardiovascular outcomes, whereas increased HRV has cardioprotective effects (82). Hinton et al. (148) found that after intervention with *γ*-aminobutyric acid (GABA)-enriched oolong tea in 30 subjects, autonomic nervous stability and HRV improved, concluding that GABA-enriched oolong tea has beneficial effects on HRV and autonomic nervous function. However, due to the inherent function of GABA and the lack of further mechanistic studies, it remains unclear whether these effects are due to GABA, oolong tea, or their combined action (83).

## 4 Intervention strategies

**Figure 5:**
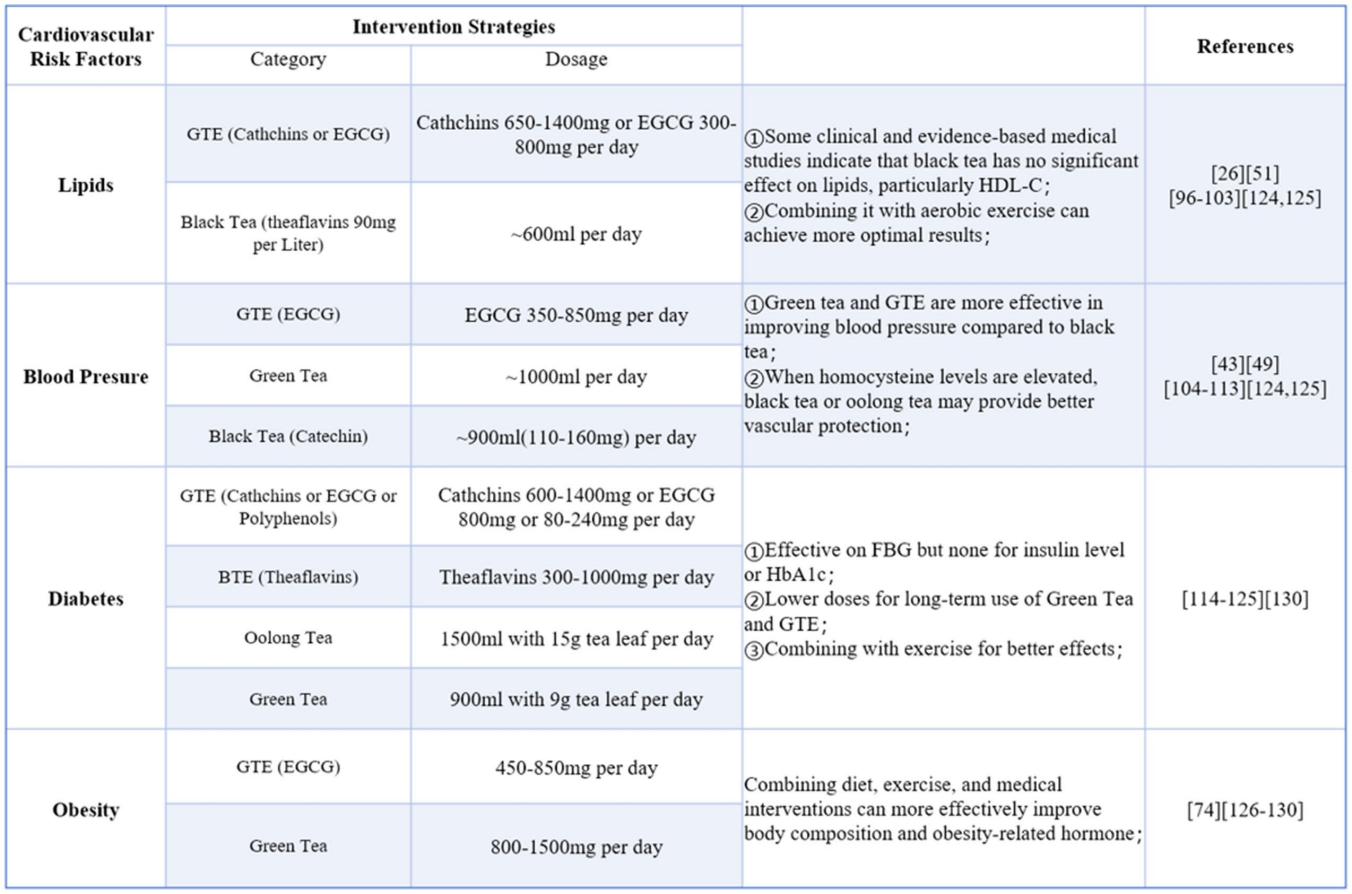
Intervention strategies for cardiovascular risk factors (Green tea extra-GTE, Black tea extra-BTE).

The intervention strategies section was developed by setting keywords such as tea, tea extracts, tea components, cardiovascular risk factors, blood glucose, blood lipids, blood pressure, and obesity for searches in PubMed and Web of Science. The document type was limited to clinical studies, systematic reviews, and meta-analyses published between 2010 and 2025. Animal and cell research were excluded from the collected literature. Based on the results of the collected studies, tea intervention strategies were proposed for different cardiovascular risk factors (Figure 5).

In some systematic reviews and meta-analyses, the publication dates of the included studies were relatively early and the intervention dosages maybe unreasonable. To ensure the safety and scientific validity of the recommended strategies, the application dosages also referenced the tea safety standards outlined in the MOFCOM-published “*Technical Guide to Export Commodities.”*

## 5 Discussion

This review summarizes the characteristics, chemical components, potential cardiovascular benefits, and possible mechanisms of tea. It also proposes the vision of utilizing tea as a nutritional intervention in primary and secondary prevention of cardiovascular diseases (CVDs). On one hand, tea is a widely consumed soft drink with low cost, offering good economic value, and numerous studies have confirmed the safety of tea interventions in patients with cardiovascular diseases. On the other hand, tea is rich in bioactive compounds such as tea polyphenols, catechins, theanine, and tea polysaccharides. These compounds exhibit multiple effects, including lowering blood lipids, reducing blood pressure, improving diabetes, and providing antioxidant and anti-inflammatory benefits. These attributes align with the needs of primary and secondary prevention of CVDs, making tea a promising choice for nutritional intervention (84–86).

Current tea-related research predominantly focuses on green tea, black tea, and individual component extracts, the former primarily due to their high catechin or theaflavin content, and the latter due to the purity of the extracted components, which facilitates the study of specific bioactivities and mechanisms. Although the application of tea infusions or extracts in studies has increased, better reflecting daily tea consumption, most studies cannot explicitly identify the precise source of tea’s therapeutic effects on diseases or symptoms. This may be due to the complex and diverse bioactive components in tea leaves and the still incompletely understood mechanisms of their actions (87).

The chemical composition differences caused by various tea varieties and processing methods add complexity to the research. Therefore, despite the widespread recognition of the health benefits of green and black tea, further in-depth research and exploration are required to clarify tea’s therapeutic effects on specific diseases or symptoms. The positive effects of tea components on various diseases are the main focus of most health-related tea research. Chen et al. (149) demonstrated ideal anti-inflammatory effects by inducing the production of nitric oxide, iNOS, COX-2, IL-6, and IL-10 in Raw 264.7 cells using processing techniques of green and black tea applied to coffee leaves. This indicates that the positive effects of tea on human health may not only come from its inherent chemical components but also be influenced by processing methods that alter the content and proportion of active ingredients in tea leaves (88).

The FAO report on tea consumption shows that global tea production and consumption continue to grow, driven by an increasing awareness of tea’s health benefits, with black tea and dark tea being the fastest-growing types, primarily in China and other Asian countries (89). Additionally, due to the growth environment and production methods of tea largely originating in Asia and the relatively high number of related studies, research results can vary by region and population (90). For example, the dose–response relationship between green tea consumption and CAD risk shows a significant negative correlation between cardiovascular disease risk and green tea consumption in Asian populations, while this correlation is not observed in Western populations. It was found that an additional cup of tea per day is associated with a 4% reduction in CVD mortality, a 2% reduction in CVD events, and a 1.5% reduction in all-cause mortality, with higher correlations in elderly individuals (91, 92). This situation highlights the lack of broad representativeness in the impact of tea on cardiovascular risk factors. Future research needs to expand the study’s geographic scope and include subjects from different racial groups for further exploration.

## References

[1] Visseren FLJ Mach F Smulders YM Carballo D Koskinas KC Bäck M. ESC scientific document group. 2021 ESC guidelines on cardiovascular disease prevention in clinical practice. Eur J Prev Cardiol. (2022) 29:5–115. doi: 10.1093/eurjpc/zwab154 34558602

[2] McPhee PG Singh S Morrison KM. Childhood obesity and cardiovascular disease risk: working toward solutions. Can J Cardiol. (2020) 36:1352–61. doi: 10.1016/j.cjca.2020.06.020, PMID: 32622878

[3] Korczak DJ Cleverley K Birken CS Pignatiello T Mahmud FH McCrindle BW. Cardiovascular disease risk factors among children and adolescents with depression. Front Psych. (2021) 12:702737. doi: 10.3389/fpsyt.2021.702737 PMC8418089 34489758

[4] Qiu L Wang W Sa R Liu F. Prevalence and risk factors of hypertension, diabetes, and dyslipidemia among adults in Northwest China. Int J Hypertens. (2021) 2021:1–10. doi: 10.1155/2021/5528007, PMID: 33936811 PMC8055385

[5] Paquette M Bernard S Paré G Baass A. Triglycerides, hypertension, and smoking predict cardiovascular disease in dysbetalipoproteinemia. J Clin Lipidol. (2020) 14:46–52. doi: 10.1016/j.jacl.2019.12.006, PMID: 31959563

[6] Luo Q Luo L Zhao J Wang Y Luo H. Biological potential and mechanisms of Tea's bioactive compounds: an updated review. J Adv Res. (2023) S2090-1232:00378. doi: 10.1016/j.jare.2023.12.004 PMC11519742 38056775

[7] Rajendran A Minhas AS Kazzi B Varma B Choi E Thakkar A. Sex-specific differences in cardiovascular risk factors and implications for cardiovascular disease prevention in women. Atherosclerosis. (2023) 384:117269. doi: 10.1016/j.atherosclerosis.2023.117269 37752027 PMC10841060

[8] Teo KK Rafiq T. Cardiovascular risk factors and prevention: a perspective from developing countries. Can J Cardiol. (2021) 37:733–43. doi: 10.1016/j.cjca.2021.02.009, PMID: 33610690

[9] Keller A Wallace TC. Tea intake and cardiovascular disease: an umbrella review. Ann Med. (2021) 53:929–44. doi: 10.1080/07853890.2021.1933164, PMID: 34396859 PMC8366653

[10] Tao W Zhou Z Zhao B Wei T. Simultaneous determination of eight catechins and four theaflavins in green, black and oolong tea using new HPLC-MS-MS method. J Pharm Biomed Anal. (2016) 131:140–5. doi: 10.1016/j.jpba.2016.08.020, PMID: 27589031

[11] Wang W Liu K Dong H Liao W Yang X He Q. A frontier exploration of ancient craftsmanship: effects of various tea products in traditional Chinese cuisine "tea flavored beef". Food Chem. (2024) 454:139834. doi: 10.1016/j.foodchem.2024.139834, PMID: 38815322

[12] Khan N Mukhtar H. Tea polyphenols in promotion of human health. Nutrients. (2018) 11:39. doi: 10.3390/nu11010039, PMID: 30585192 PMC6356332

[13] Recent insights into the physicochemical properties. Bioactivities and their relationship of tea polysaccharides. Food Chem. (2024) 432:137223. doi: 10.1016/j.foodchem.2023.137223, PMID: 37669580

[14] Powell-Wiley TM Poirier P Burke LE Després JP Gordon-Larsen P Lavie CJ. American Heart Association Council on lifestyle and Cardiometabolic health; council on cardiovascular and stroke nursing; council on clinical cardiology; council on epidemiology and prevention; and stroke council. obesity and cardiovascular disease: a scientific statement from the American Heart Association. Circulation. (2021) 143:e984–e1010. doi: 10.1161/CIR.0000000000000973, PMID: 33882682 PMC8493650

[15] Tran HH Mansoor M Butt SRR Satnarine T Ratna P Sarker A. Impact of green tea consumption on the prevalence of cardiovascular outcomes: a systematic review. Cureus. (2023) 15:e49775. doi: 10.7759/cureus.49775, PMID: 38161525 PMC10757748

[16] Fang Y Wang J Cao Y Liu W Duan L Hu J. The Antiobesity effects and potential mechanisms of Theaflavins. J Med Food. (2024) 27:1–11. doi: 10.1089/jmf.2023.K.0180, PMID: 38060708

[17] Han LK Takaku T Li J Kimura Y Okuda H. Anti-obesity action of oolong tea. Int J Obes Relat Metab Disord. (1999) 23:98–105. PMID: 10094584 10.1038/sj.ijo.0800766

[18] Zhang S Takano J Murayama N Tominaga M Abe T Park I. Subacute ingestion of caffeine and oolong tea increases fat oxidation without affecting energy expenditure and sleep architecture: a randomized, placebo-controlled, double-blinded cross-over trial. Nutrients. (2020) 12:3671. doi: 10.3390/nu12123671, PMID: 33260552 PMC7760339

[19] Alipour B Rashidkhani B Edalati S. Dietary flavonoid intake, total antioxidant capacity and lipid oxidative damage: a cross-sectional study of Iranian women. Nutrition. (2016) 32:566–72. doi: 10.1016/j.nut.2015.11.011, PMID: 26830011

[20] Raederstorff DG Schlachter MF Elste V Weber P. Effect of EGCG on lipid absorption and plasma lipid levels in rats. J Nutr Biochem. (2003) 14:326–32. doi: 10.1016/S0955-2863(03)00054-8, PMID: 12873714

[21] Huang F Zheng X Ma X Jiang R Zhou W Zhou S. Theabrownin from Pu-erh tea attenuates hypercholesterolemia via modulation of gut microbiota and bile acid metabolism. Nat Commun. (2019) 10:4971. doi: 10.1038/s41467-019-12896-x, PMID: 31672964 PMC6823360

[22] Kashif M Imran A Saeed F Chatha SAS Arshad MU. Catechins, theaflavins and ginger freeze-dried extract based functional drink significantly mitigate the hepatic, diabetic and lipid abnormalities in rat model. Cell Mol Biol (Noisy-le-Grand). (2021) 67:132–41. doi: 10.14715/cmb/2021.67.1.20, PMID: 34817356

[23] Fu H He J Li C Chang H. Theaflavin-3,3'-Digallate protects liver and kidney functions in diabetic rats by up-regulating Circ-ITCH and Nrf2 signaling pathway. J Agric Food Chem. (2024) 72:14630–9. doi: 10.1021/acs.jafc.3c08251, PMID: 38634619

[24] Wang Y Xia H Yu J Sui J Pan D Wang S. Effects of green tea catechin on the blood pressure and lipids in overweight and obese population-a meta-analysis. Heliyon. (2023) 9:e21228. doi: 10.1016/j.heliyon.2023.e21228, PMID: 38034724 PMC10681946

[25] Hematinezhad Touli M Elmieh A Hosseinpour A. The effect of six-week aerobic exercise combined with ease max Incr2Green tea consumption on PON1 and VOand Apelin, blood pressure, and blood lipids reduction in young obese men. Arch Razi Inst. (2022) 77:2115–23. doi: 10.22092/ARI.2022.357847.2109, PMID: 37274908 PMC10237557

[26] Trautwein EA Du Y Meynen E Yan X Wen Y Wang H. Purified black tea theaflavins and theaflavins/catechin supplements did not affect serum lipids in healthy individuals with mildly to moderately elevated cholesterol concentrations. Eur J Nutr. (2010) 49:27–35. doi: 10.1007/s00394-009-0045-7, PMID: 19639377

[27] Quezada-Fernández P Trujillo-Quiros J Pascoe-González S Trujillo-Rangel WA Cardona-Müller D Ramos-Becerra CG. Effect of green tea extract on arterial stiffness, lipid profile and sRAGE in patients with type 2 diabetes mellitus: a randomised, double-blind, placebo-controlled trial. Int J Food Sci Nutr. (2019) 70:977–85. doi: 10.1080/09637486.2019.1589430, PMID: 31084381

[28] Scolaro B Nogueira MS Paiva A Bertolami A Barroso LP Vaisar T. Statin dose reduction with complementary diet therapy: a pilot study of personalized medicine. Mol Metab. (2018) 11:137–44. doi: 10.1016/j.molmet.2018.02.005, PMID: 29503145 PMC6001350

[29] Katada S Oishi S Yanagawa K Ishii S Oki M Matsui Y. Concomitant use of tea catechins affects absorption and serum triglyceride-lowering effects of monoglucosyl hesperidin. Food Funct. (2021) 12:9339–46. doi: 10.1039/D1FO01917A, PMID: 34606551

[30] Doyle AE. Hypertension and vascular disease. Am J Hypertens. (1991) 4:103S–6S. doi: 10.1093/ajh/4.2.103S, PMID: 2021454

[31] Hodgson JM Devine A Puddey IB Chan SY Beilin LJ Prince RL. Tea intake is inversely related to blood pressure in older women. J Nutr. (2003) 133:2883–6. doi: 10.1093/jn/133.9.2883, PMID: 12949382

[32] Yang YC Lu FH Wu JS Wu CH Chang CJ. The protective effect of habitual tea consumption on hypertension. Arch Intern Med. (2004) 164:1534–40. doi: 10.1001/archinte.164.14.1534, PMID: 15277285

[33] Dludla PV Nkambule BB Mazibuko-Mbeje SE Nyambuya TM Orlando P Silvestri S. Tea consumption and its effects on primary and secondary prevention of coronary artery disease: qualitative synthesis of evidence from randomized controlled trials. Clin Nutr ESPEN. (2021) 41:77–87. doi: 10.1016/j.clnesp.2020.11.006, PMID: 33487310

[34] Miller RJ Jackson KG Dadd T Mayes AE Brown AL Minihane AM. The impact of the catechol-O-methyltransferase genotype on the acute responsiveness of vascular reactivity to a green tea extract. Br J Nutr. (2011) 105:1138–44. doi: 10.1017/S0007114510004836, PMID: 21144097

[35] Hodgson JM Burke V Puddey IB. Acute effects of tea on fasting and postprandial vascular function and blood pressure in humans. J Hypertens. (2005) 23:47–54. doi: 10.1097/00004872-200501000-00012 PMID: 15643124

[36] Tinelli C Di Pino A Ficulle E Marcelli S Feligioni M. Hyperhomocysteinemia as a risk factor and potential nutraceutical target for certain pathologies. Front Nutr. (2019) 6:49. doi: 10.3389/fnut.2019.00049, PMID: 31069230 PMC6491750

[37] Persson M Josefsson KP Andersson RG. Tea flavanols inhibit angiotensin converting enzyme activity and increase nitric oxide production in human endothelial cells. JPharm Pharmacol. (2006) 58:1139–44. doi: 10.1211/jpp.58.8.0016, PMID: 16872562

[38] Rufian-Henares JA Morales FJ. Angiotensin-I converting enzyme inhibitory activity of coffee melanoidins. J Agric Food Chem. (2007) 55:1480–5. doi: 10.1021/jf062604d, PMID: 17243703

[39] Hodgson JM. Effects of tea and tea flavonoids on endothelial function and blood pressure: a brief review. Clin Exp Pharmacol Physiol. (2006) 33:838–41. doi: 10.1111/j.1440-1681.2006.04450.x, PMID: 16922817

[40] Wang L Tian X. Epigallocatechin-3-Gallate protects against homocysteine-induced brain damage in rats. Planta Med. (2018) 84:34–41. doi: 10.1055/s-0043-114865, PMID: 28666294

[41] Pei J Guo S Zhang C Xie K. Influence of tea polyphenols on the damage of fibrinolytic system induced by homocysteine in human umbilical vein endothelial cells. Wei Sheng Yan Jiu. (2009) 38:47–50. Chinese. doi: 10.19813/j.cnki.weishengyanjiu.2009.01.015 PMID: 19267075

[42] Lee WJ Shim JY Zhu BT. Mechanisms for the inhibition of DNA methyltransferases by tea catechins and bioflavonoids. Mol Pharmacol. (2005) 68:1018–30. doi: 10.1124/mol.104.008367, PMID: 16037419

[43] Zhu J Wang W Xiong Y Cooper RS Du Raza-Arvizu R Cao G. The association between tea consumption and Hyperhomocysteine in Chinese hypertensive patients. Am J Hypertens. (2019) 32:209–15. doi: 10.1093/ajh/hpy163, PMID: 30379988

[44] Wang W Sun Y Liu J Wang J Li Y Li H. Protective effect of theaflavins on homocysteine-induced injury in HUVEC cells in vitro. J Cardiovasc Pharmacol. (2012) 59:434–40. doi: 10.1097/FJC.0b013e318248aeb3, PMID: 22217883

[45] Hodgson JM Burke V Beilin LJ Croft KD Puddey IB. Can black tea influence plasma total homocysteine concentrations? Am J Clin Nutr. (2003) 77:907–11. doi: 10.1093/ajcn/77.4.907, PMID: 12663290

[46] Dos Passos RR Santos CV Priviero F Briones AM Tostes RC Webb RC. Immunomodulatory activity of cytokines in hypertension: a vascular perspective. Hypertension. (2024) 81:1411–23. doi: 10.1161/HYPERTENSIONAHA.124.21712, PMID: 38686582 PMC11168883

[47] Liang Y Ip MSM Mak JCW. (−)-Epigallocatechin-3-gallate suppresses cigarette smoke-induced inflammation in human cardiomyocytes via ROS-mediated MAPK and NF-κB pathways. Phytomedicine. (2019) 58:152768. doi: 10.1016/j.phymed.2018.11.028 31005721

[48] Reddy AT Lakshmi SP Maruthi Prasad E Varadacharyulu NC Kodidhela LD. Epigallocatechin gallate suppresses inflammation in human coronary artery endothelial cells by inhibiting NF-κB. Life Sci. (2020) 258:118136. doi: 10.1016/j.lfs.2020.118136, PMID: 32726662

[49] Mahdavi-Roshan M Salari A Ghorbani Z Ashouri A. The effects of regular consumption of green or black tea beverage on blood pressure in those with elevated blood pressure or hypertension: a systematic review and meta-analysis. Complement Ther Med. (2020) 51:102430. doi: 10.1016/j.ctim.2020.102430, PMID: 32507441

[50] Biesinger S Michaels HA Quadros AS Qian Y Rabovsky AB Badger RS. A combination of isolated phytochemicals and botanical extracts lowers diastolic blood pressure in a randomized controlled trial of hypertensive subjects. Eur J Clin Nutr. (2016) 70:10–6. doi: 10.1038/ejcn.2015.88, PMID: 26059745

[51] Nogueira LP Nogueira Neto JF Klein MR Sanjuliani AF. Short-term effects of green tea on blood pressure, endothelial function, and metabolic profile in obese Prehypertensive women: a crossover randomized clinical trial. J Am Coll Nutr. (2017) 36:108–15. doi: 10.1080/07315724.2016.1194236, PMID: 27797683

[52] Sattar N Rawshani A Franzén S Rawshani A Svensson AM Rosengren A. Age at diagnosis of type 2 diabetes mellitus and associations with cardiovascular and mortality risks. Circulation. (2019) 139:2228–37. doi: 10.1161/circulationaha.118.037885, PMID: 30955347

[53] Rawshani A Sattar N Franzen S Rawshani A Hattersley AT Svensson AM. Excess mortality and cardiovascular disease in young adults with type 1 diabetes in relation to age at onset: a nationwide, register-based cohort study. Lancet. (2018) 392:477486:477–86. doi: 10.1016/S0140-6736(18)31506-X, PMID: 30129464 PMC6828554

[54] Rawshani A Rawshani A Sattar N Franzen S McGuire DK Eliasson B. Relative prognostic importance and optimal levels of risk factors for mortality and cardiovascular outcomes in type 1 diabetes mellitus. Circulation. (2019) 139:19001912.doi: 10.1161/CIRCULATIONAHA.118.037454 30798638

[55] Livingstone SJ Looker HC Hothersall EJ Wild SH Lindsay RS Chalmers J. Risk of cardiovascular disease and total mortality in adults with type 1 diabetes: Scottish registry linkage study. PLoSMed. (2012) 9:e1001321. doi: 10.1371/journal.pmed.1001321, PMID: 23055834 PMC3462745

[56] Liu B Gu S Zhang J Zhou H Su J Wang S. Green tea consumption and incidence of cardiovascular disease in type 2 diabetic patients with overweight/obesity: a community-based cohort study. Arch Public Health. (2024) 82:18. doi: 10.1186/s13690-024-01242-3, PMID: 38308353 PMC10835928

[57] Mi Y Liu X Tian H Liu H Li J Qi G. EGCG stimulates the recruitment of brite adipocytes, suppresses adipogenesis and counteracts TNF-α-triggered insulin resistance in adipocytes. Food Funct. (2018) 9:3374–86. doi: 10.1039/C8FO00167G, PMID: 29868672

[58] Ren Z Yang Z Lu Y Zhang R Yang H. Anti-glycolipid disorder effect of epigallocatechin-3-gallate on high-fat diet and STZ-induced T2DM in mice. Mol Med Rep. (2020) 21:2475–83. doi: 10.3892/mmr.2020.11041, PMID: 32236613 PMC7185284

[59] Esposito F Pala N Carcelli M Boateng ST D'Aquila PS Mariani A. α-Glucosidase inhibition by green, white and oolong teas: in vitro activity and computational studies. J Enzyme Inhib Med Chem. (2023) 38:2236802. doi: 10.1080/14756366.2023.2236802, PMID: 37470394 PMC10361001

[60] Liu J Ding H Yan C He Z Zhu H Ma KY. Effect of tea catechins on gut microbiota in high fat diet-induced obese mice. J Sci Food Agric. (2023) 103:2436–45. doi: 10.1002/jsfa.12476, PMID: 36715435

[61] Ni D Ai Z Munoz-Sandoval D Suresh R Ellis PR Yuqiong C. Inhibition of the facilitative sugar transporters (GLUTs) by tea extracts and catechins. FASEB J. (2020) 34:9995–10010. doi: 10.1096/fj.202000057RR, PMID: 32564472

[62] Zeng M Hodges JK Pokala A Khalafi M Sasaki GY Pierson J. A green tea extract confection decreases circulating endotoxin and fasting glucose by improving gut barrier function but without affecting systemic inflammation: a double-blind, placebo-controlled randomized trial in healthy adults and adults with metabolic syndrome. Nutr Res. (2024) 124:94–110. doi: 10.1016/j.nutres.2024.02.001, PMID: 38430822

[63] Liang Y Wu F Wu D Zhu X Gao X Hu X. Fu loose tea administration ameliorates obesity in high-fat diet-fed C57BL/6J mice: a comparison with Fu brick tea and orlistat. Food Secur. (2024) 13:206. doi: 10.3390/foods13020206, PMID: 38254507 PMC10815023

[64] Wang R Gu M Zhang Y Zhong Q Chen L Li D. Long-term drinking of green tea combined with exercise improves hepatic steatosis and obesity in male mice induced by high-fat diet. Food Sci Nutr. (2023) 12:776–85. doi: 10.1002/fsn3.3773, PMID: 38370081 PMC10867457

[65] Tong T Ren N Soomi P Wu J Guo N Kang H. Theaflavins improve insulin sensitivity through regulating mitochondrial biosynthesis in palmitic acid-induced HepG2 cells. Molecules. (2018) 23:3382. 30572687 10.3390/molecules23123382 PMC6320999

[66] Al-Rawaf HA Gabr SA Alghadir AH. Potential roles of circulating microRNAs in the healing of type 1 diabetic wounds treated with green tea extract: molecular and biochemical study. Heliyon. (2023) 9:e22020. doi: 10.1016/j.heliyon.2023.e22020, PMID: 38027999 PMC10665742

[67] Seifalian AM Filippatos TD Joshi J Mikhailidis DP. Obesity and arterial compliance alterations. Curr Vasc Pharmacol. (2010) 8:155–68. doi: 10.2174/157016110790886956, PMID: 20180777

[68] Zarzour A Kim HW Weintraub NL. Understanding obesity-related cardiovascular disease: It's all about balance. Circulation. (2018) 138:64–6. doi: 10.1161/circulationaha.118.034454, PMID: 29967231 PMC6053309

[69] Rosengren A Hawken S Ounpuu S Sliwa K Zubaid M Almahmeed WA. Association of psychosocial risk factors with risk of acute myocardial infarction in 11119 cases and 13648 controls from 52 countries (the INTERHEART study): case-control study. Lancet. (2004) 364:953962:953–62. doi: 10.1016/S0140-6736(04)17019-0, PMID: 15364186

[70] Vaccarino V Badimon L Bremner JD Cenko E Cubedo J Dorobantu M. Position paper of the ESC working group on coronary pathophysiology and microcirculation. Eur Heart J. (2018) 2020:16871696. doi: 10.1093/eurheartj/ehy913 PMC10941327 30698764

[71] Nederveen JP Mastrolonardo AJ Xhuti D Di Carlo A Manta K Fuda MR. Novel multi-ingredient supplement facilitates weight loss and improves body composition in overweight and obese individuals: a randomized, double-blind, placebo-controlled clinical trial. Nutrients. (2023) 15:3693. doi: 10.3390/nu15173693, PMID: 37686725 PMC10490028

[72] Xie L Tang Q Yao D Gu Q Zheng H Wang X. Effect of decaffeinated green tea polyphenols on body fat and precocious puberty in obese girls: a randomized controlled trial. Front Endocrinol. (2021) 12:736724., PMID: 34712203 10.3389/fendo.2021.736724 PMC8546255

[73] Asbaghi O Rezaei Kelishadi M Larky DA Bagheri R Amirani N Goudarzi K. The effects of green tea extract supplementation on body composition, obesity-related hormones and oxidative stress markers: a grade-assessed systematic review and dose-response meta-analysis of randomised controlled trials. Br J Nutr. (2024) 131:1125–57. doi: 10.1017/S000711452300260X, PMID: 38031409

[74] Dulloo AG Seydoux J Girardier L Chantre P Vandermander J. Green tea and thermogenesis: interactions between catechin-polyphenols, caffeine and sympathetic activity. Int J Obes Relat Metab Disord. (2000) 24:252–8. doi: 10.1038/sj.ijo.0801101, PMID: 10702779

[75] Tung YC Liang ZR Yang MJ Ho CT Pan MH. Oolong tea extract alleviates weight gain in high-fat diet-induced obese rats by regulating lipid metabolism and modulating gut microbiota. Food Funct. (2022) 13:2846–56. doi: 10.1039/D1FO03356E, PMID: 35179170

[76] Xie L Tang Q Yao D Gu Q Zheng H Wang X. Effect of decaffeinated green tea polyphenols on body fat and Precocio us puberty in obese girls: a randomized controlled trial. Front Endocrinol. (2021) 12:736724. doi: 10.3389/fendo.2021.736724 PMC8546255 34712203

[77] Braun J Patel M Kameneva T Keatch C Lambert G Lambert E. Central stress pathways in the development of cardiovascular disease. Clin Auton Res. (2023) 34:99–116. doi: 10.1007/s10286-023-01008-x, PMID: 38104300

[78] Bruno RM Ghiadoni L. Polyphenols, antioxidants and the sympathetic nervous system. Curr Pharm Des. (2018) 24:130–9. doi: 10.2174/1381612823666171114170642, PMID: 29141540

[79] Garcia ML Pontes RB Nishi EE Ibuki FK Oliveira V Sawaya AC. The antioxidant effects of green tea reduces blood pressure and sympathoexcitation in an experimental model of hypertension. J Hypertens. (2017) 35:348–54. doi: 10.1097/HJH.0000000000001149, PMID: 28005704

[80] Sanae F Miyaichi Y Kizu H Hayashi H. Effects of catechins on vascular tone in rat thoracic aorta with endothelium. Life Sci. (2002) 71:2553–62. doi: 10.1016/S0024-3205(02)02080-5, PMID: 12270760

[81] Cygankiewicz I Zareba W. Heart rate variability. Handb Clin Neurol. (2013) 117:379–93. doi: 10.1016/B978-0-444-53491-0.00031-6, PMID: 24095141

[82] Rajendra Acharya U Paul Joseph K Kannathal N Lim CM Suri JS. Heart rate variability: a review. Med Biol Eng Comput. (2006) 44:1031–51. doi: 10.1007/s11517-006-0119-0 PMID: 17111118

[83] Hinton T Jelinek HF Viengkhou V Johnston GA Matthews S. Effect of GABA-fortified oolong tea on reducing stress in a university student cohort. Front Nutr. (2019) 6:27. doi: 10.3389/fnut.2019.00027, PMID: 30972340 PMC6443991

[84] Sagris M Vlachakis PK Simantiris S Theofilis P Gerogianni M Karakasis P. From a cup of tea to cardiovascular care: vascular mechanisms of action. Life. (2024) 14:1168. doi: 10.3390/life14091168, PMID: 39337950 PMC11433009

[85] American Association of Cardiovascular & Pulmonary Rehabilitation. Guidelines for cardiac rehabilitation and secondary prevention programs. US: Promoting Health & Preventing Disease Human Kinetics Publishers (1999).

[86] Thomas RJ Huang HH. Cardiac rehabilitation for secondary prevention of cardiovascular disease: 2019 update. Curr Treat Options Cardiovasc Med. (2019) 21:56. doi: 10.1007/s11936-019-0759-7, PMID: 31486974

[87] Ho C-T Lin J-K Shahidi F. Tea and tea products: Chemistry and health-promoting properties. 1st ed. Boca Raton: CRC Press (2008).

[88] Chen X Mu K Kitts DD. Characterization of phytochemical mixtures with inflammatory modulation potential from coffee leaves processed by green and black tea processing methods. Food Chem. (2019) 271:248–58. doi: 10.1016/j.foodchem.2018.07.097, PMID: 30236674

[89] United Nations FAO. International tea market: Market situation, prospects and emerging issues. (2022). Available at: https://openknowledge.fao.org/server/api/core/bitstreams/e1d8588a-ddba-4b49-9897-311611391a76/content

[90] Peters U Poole C Arab L. Does tea affect cardiovascular disease? A meta-analysis. Am J Epidemiol. (2001) 154:495–503. doi: 10.1093/aje/154.6.495, PMID: 11549554

[91] Yang X Dai H Deng R Zhang Z Quan Y Giri M. Association between tea consumption and prevention of coronary artery disease: a systematic review and dose-response meta-analysis. Front Nutr. (2022) 9:1021405. doi: 10.3389/fnut.2022.1021405, PMID: 36505265 PMC9729734

[92] Chung M Zhao N Wang D Shams-White M Karlsen M Cassidy A. Dose-response relation between tea consumption and risk of cardiovascular disease and all-cause mortality: a systematic review and Meta-analysis of population-based studies. Adv Nutr. (2020) 11:790–814. doi: 10.1093/advances/nmaa010, PMID: 32073596 PMC7360449

[93] Tang GY Zhao CN Xu XY Gan RY Cao SY Liu Q. Phytochemical composition and antioxidant capacity of 30 Chinese teas. Antioxidants. (2019) 8:180. doi: 10.3390/antiox8060180, PMID: 31216700 PMC6617242

[94] Mirza O Henriksen A Ostergaard L Welinder KG Gajhede M. *Arabidopsis thaliana* peroxidase N: structure of a novel neutral peroxidase. Acta Crystallogr D Biol Crystallogr. (2000) 56:372–5. doi: 10.1107/S0907444999016340, PMID: 10713531

[95] Putnam CD Arvai AS Bourne Y Tainer JA. Active and inhibited human catalase structures: ligand and NADPH binding and catalytic mechanism. J Mol Biol. (2000) 296:295–309. PMID: 10656833 10.1006/jmbi.1999.3458

[96] Yuan F Dong H Fang K Gong J Lu F. Effects of green tea on lipid metabolism in overweight or obese people: a meta-analysis of randomized controlled trials. Mol Nutr Food Res. (2018) 62. doi: 10.1002/mnfr.201601122, PMID: 28636182

[97] Xu R Yang K Li S Dai M Chen G. Effect of green tea consumption on blood lipids: a systematic review and meta-analysis of randomized controlled trials. Nutr J. (2020) 19:48. doi: 10.1186/s12937-020-00557-5, PMID: 32434539 PMC7240975

[98] Li A Wang Q Li P Zhao N Liang Z. Effects of green tea on lipid profile in overweight and obese women. Int J Vitam Nutr Res. (2024) 94:239–51. doi: 10.1024/0300-9831/a000783, PMID: 37082776

[99] Kim A Chiu A Barone MK Avino D Wang F Coleman CI. Green tea catechins decrease total and low-density lipoprotein cholesterol: a systematic review and meta-analysis. J Am Diet Assoc. (2011) 111:1720–9. doi: 10.1016/j.jada.2011.08.009, PMID: 22027055

[100] Bahorun T Luximon-Ramma A Neergheen-Bhujun VS Gunness TK Googoolye K Auger C. The effect of black tea on risk factors of cardiovascular disease in a normal population. Prev Med. (2012) 54:S98–S102. doi: 10.1016/j.ypmed.2011.12.009, PMID: 22198621

[101] Macêdo APA Gonçalves MDS Barreto Medeiros JM David JM Villarreal CF Macambira SG. Potential therapeutic effects of green tea on obese lipid profile - a systematic review. Nutr Health. (2022) 28:401–15. doi: 10.1177/02601060211073236, PMID: 35014893

[102] Araya-Quintanilla F Gutiérrez-Espinoza H Moyano-Gálvez V Muñoz-Yánez MJ Pavez L García K. Effectiveness of black tea versus placebo in subjects with hypercholesterolemia: a PRISMA systematic review and meta-analysis. Diabetes Metab Syndr. (2019) 13:2250–8. doi: 10.1016/j.dsx.2019.05.019, PMID: 31235165

[103] Wang D Chen C Wang Y Liu J Lin R. Effect of black tea consumption on blood cholesterol: a meta-analysis of 15 randomized controlled trials. PLoS One. (2014) 9:e107711. doi: 10.1371/journal.pone.0107711, PMID: 25237889 PMC4169558

[104] Liu G Mi XN Zheng XX Xu YL Lu J Huang XH. Effects of tea intake on blood pressure: a meta-analysis of randomised controlled trials. Br J Nutr. (2014) 112:1043–54. doi: 10.1017/S0007114514001731, PMID: 25137341

[105] Li G Zhang Y Thabane L Mbuagbaw L Liu A Levine MA. Effect of green tea supplementation on blood pressure among overweight and obese adults: a systematic review and meta-analysis. J Hypertens. (2015) 33:243–54. doi: 10.1097/HJH.0000000000000426, PMID: 25479028

[106] Yarmolinsky J Gon G Edwards P. Effect of tea on blood pressure for secondary prevention of cardiovascular disease: a systematic review and meta-analysis of randomized controlled trials. Nutr Rev. (2015) 73:236–46. doi: 10.1093/nutrit/nuv001 26024546

[107] Xu R Yang K Ding J Chen G. Effect of green tea supplementation on blood pressure: a systematic review and meta-analysis of randomized controlled trials. Medicine. (2020) 99:e19047. doi: 10.1097/MD.0000000000019047, PMID: 32028419 PMC7015560

[108] Onakpoya I Spencer E Heneghan C Thompson M. The effect of green tea on blood pressure and lipid profile: a systematic review and meta-analysis of randomized clinical trials. Nutr Metab Cardiovasc Dis. (2014) 24:823–36. doi: 10.1016/j.numecd.2014.01.016, PMID: 24675010

[109] Grassi D Draijer R Desideri G Mulder T Ferri C. Black tea lowers blood pressure and wave reflections in fasted and postprandial conditions in hypertensive patients: a randomised study. Nutrients. (2015) 7:1037–51. doi: 10.3390/nu7021037, PMID: 25658240 PMC4344573

[110] Al-Shafei AIM El-Gendy OAA. Regular consumption of green tea improves pulse pressure and induces regression of left ventricular hypertrophy in hypertensive patients. Physiol Rep. (2019) 7:e14030. doi: 10.14814/phy2.14030, PMID: 30912296 PMC6434072

[111] Quan J Zhang T Gu Y Meng G Zhang Q Liu L. Green tea intake and the risk of hypertension in premenopausal women: the TCLSIH cohort study. Food Funct. (2023) 14:4406–13. doi: 10.1039/D2FO03342A, PMID: 37097224

[112] Khalesi S Sun J Buys N Jamshidi A Nikbakht-Nasrabadi E Khosravi-Boroujeni H. Green tea catechins and blood pressure: a systematic review and meta-analysis of randomised controlled trials. Eur J Nutr. (2014) 53:1299–311. doi: 10.1007/s00394-014-0720-1, PMID: 24861099

[113] Lin CC Hsieh CY Chen LF Chen YC Ho TH Chang SC. Versatile effects of GABA oolong tea on improvements in diastolic blood pressure, alpha brain waves, and quality of life. Food Secur. (2023) 12:4101. doi: 10.3390/foods12224101, PMID: 38002159 PMC10670354

[114] Jia MJ Liu XN Liang YC Liu DL Li HL. The effect of green tea on patients with type 2 diabetes mellitus: a meta-analysis. Medicine (Baltimore). (2024) 103:e39702. doi: 10.1097/MD.0000000000039702, PMID: 39809182 PMC11596636

[115] Li X Wang W Hou L Wu H Wu Y Xu R. Does tea extract supplementation benefit metabolic syndrome and obesity? A systematic review and meta-analysis. Clin Nutr. (2020) 39:1049–58. doi: 10.1016/j.clnu.2019.05.019, PMID: 31174941

[116] Li Y Wang C Huai Q Guo F Liu L Feng R. Effects of tea or tea extract on metabolic profiles in patients with type 2 diabetes mellitus: a meta-analysis of ten randomized controlled trials. Diabetes Metab Res Rev. (2016) 32:2–10. doi: 10.1002/dmrr.2641, PMID: 25689396

[117] Xu R Bai Y Yang K Chen G. Effects of green tea consumption on glycemic control: a systematic review and meta-analysis of randomized controlled trials. Nutr Metab. (2020) 17:56. doi: 10.1186/s12986-020-00469-5, PMID: 32670385 PMC7350188

[118] Yang CY Yen YY Hung KC Hsu SW Lan SJ Lin HC. Inhibitory effects of pu-erh tea on alpha glucosidase and alpha amylase: a systemic review. Nutr Diabetes. (2019) 9:23. doi: 10.1038/s41387-019-0092-y, PMID: 31455758 PMC6712024

[119] Momose Y Maeda-Yamamoto M Nabetani H. Systematic review of green tea epigallocatechin gallate in reducing low-density lipoprotein cholesterol levels of humans. Int J Food Sci Nutr. (2016) 67:606–13. doi: 10.1080/09637486.2016.1196655, PMID: 27324590

[120] Asbaghi O Fouladvand F Gonzalez MJ Ashtary-Larky D Choghakhori R Abbasnezhad A. Effect of green tea on glycemic control in patients with type 2 diabetes mellitus: a systematic review and meta-analysis. Diabetes Metab Syndr. (2021) 15:23–31. doi: 10.1016/j.dsx.2020.11.004, PMID: 33285391

[121] Wang X Tian J Jiang J Li L Ying X Tian H. Effects of green tea or green tea extract on insulin sensitivity and glycaemic control in populations at risk of type 2 diabetes mellitus: a systematic review and meta-analysis of randomised controlled trials. J Hum Nutr Diet. (2014) 27:501–12. doi: 10.1111/jhn.12181, PMID: 24206044

[122] He M Lyu X. Application of BRAFO-tiered approach for health benefit-risk assessment of dark tea consumption in China. Food Chem Toxicol. (2021) 158:112615. doi: 10.1016/j.fct.2021.112615, PMID: 34656696

[123] Chen S Peng D Shan Y Liu F Du R Bao Y. Black tea drinks with inulin and dextrin reduced postprandial plasma glucose fluctuations in patients with type 2 diabetes: an acute, randomized, placebo-controlled, single-blind crossover study. Nutr Diabetes. (2024) 14:95. doi: 10.1038/s41387-024-00351-w, PMID: 39616149 PMC11608310

[124] Zamani M Kelishadi MR Ashtary-Larky D Amirani N Goudarzi K Torki IA. The effects of green tea supplementation on cardiovascular risk factors: a systematic review and meta-analysis. Front Nutr. (2023) 9:1084455. doi: 10.3389/fnut.2022.1084455, PMID: 36704803 PMC9871939

[125] Neyestani TR Nikooyeh B. A comprehensive overview on the effects of green tea on anthropometric measures, blood pressure, glycemic and lipidemic status: an umbrella review and meta meta-analysis study. Nutr Metab Cardiovasc Dis. (2022) 32:2026–40. doi: 10.1016/j.numecd.2022.05.021, PMID: 35750605

[126] Zhang Y Tang N Xia W Sanjid Seraj S Pereira M Velu P. The effect of green tea supplementation on the anthropometric outcomes in overweight and obese women: a time and dose-response meta-analysis of randomized controlled trials. Crit Rev Food Sci Nutr. (2024) 64:10138–47. doi: 10.1080/10408398.2023.2220796, PMID: 37300478

[127] Lin Y Shi D Su B Wei J Găman MA Sedanur Macit M. The effect of green tea supplementation on obesity: a systematic review and dose-response meta-analysis of randomized controlled trials. Phytother Res. (2020) 34:2459–70. doi: 10.1002/ptr.6697, PMID: 32372444

[128] Baladia E Basulto J Manera M Martínez R Calbet D. Efecto del consumo de té verde o extractos de té verde en el peso y en la composición corporal; revisión sistemática y metaanálisis [effect of green tea or green tea extract consumption on body weight and body composition; systematic review and meta-analysis]. Nutr Hosp. (2014) 29:479–90. doi: 10.3305/nh.2014.29.3.7118, PMID: 24558988

[129] Vázquez Cisneros LC López-Uriarte P López-Espinoza A Navarro Meza M Espinoza-Gallardo AC Guzmán Aburto MB. Efectos del té verde y su contenido de galato de epigalocatequina (EGCG) sobre el peso corporal y la masa grasa en humanos. Una revisión sistemática [effects of green tea and its epigallocatechin (EGCG) content on body weight and fat mass in humans: a systematic review]. Nutr Hosp. (2017) 34:731–7. doi: 10.20960/nh.753, PMID: 28627214

[130] Asbaghi O Fouladvand F Gonzalez MJ Aghamohammadi V Choghakhori R Abbasnezhad A. Effect of green tea on anthropometric indices and body composition in patients with type 2 diabetes mellitus: a systematic review and Meta-analysis. Complement Med Res. (2021) 28:244–51. doi: 10.1159/000511665, PMID: 33207344

[131] Fu H He J Li C Chang H. Theaflavin-3,3’-Digallate Protects Liver and Kidney Functions in Diabetic Rats by Up-Regulating Circ-ITCH and Nrf2 Signaling Pathway. J Agric Food Chem. (2024) 72:14630–9. 38634619 10.1021/acs.jafc.3c08251

[132] Trautwein EA Du Y Meynen E Yan X Wen Y Wang H. Purified black tea theaflavins and theaflavins/catechin supplements did not affect serum lipids in healthy individuals with mildly to moderately elevated cholesterol concentrations. Eur J Nutr. (2010) 49:27–35. 19639377 10.1007/s00394-009-0045-7

[133] Quezada-Fernández P Trujillo-Quiros J Pascoe-González S Trujillo-Rangel WA Cardona-Müller D Ramos-Becerra CG. Effect of green tea extract on arterial stiffness, lipid profile and sRAGE in patients with type 2 diabetes mellitus: a randomised, double-blind, placebo-controlled trial. Int J Food Sci Nutr. (2019) 70:977–85. 31084381 10.1080/09637486.2019.1589430

[134] Scolaro B Nogueira MS Paiva A Bertolami A Barroso LP Vaisar T. Statin dose reduction with complementary diet therapy: A pilot study of personalized medicine. Mol Metab. (2018) 11:137–44. 29503145 10.1016/j.molmet.2018.02.005 PMC6001350

[135] Katada S Oishi S Yanagawa K Ishii S Oki M Matsui Y. Concomitant use of tea catechins affects absorption and serum triglyceride-lowering effects of monoglucosyl hesperidin. Food Funct. (2021) 12:9339–46. 34606551 10.1039/d1fo01917a

[136] Hodgson JM Devine A Puddey IB Chan SY Beilin LJ Prince RL. Tea intake is inversely related to blood pressure in older women. J Nutr. (2003) 133:2883–6. 12949382 10.1093/jn/133.9.2883

[137] Wang L Tian X. Epigallocatechin-3-Gallate Protects against Homocysteine-Induced Brain Damage in Rats. Planta Med. (2018) 84:34–41. 28666294 10.1055/s-0043-114865

[138] Reddy AT Lakshmi SP Maruthi Prasad E Varadacharyulu NC Kodidhela LD. Epigallocatechin gallate suppresses inflammation in human coronary artery endothelial cells by inhibiting NF-κB. Life Sci. (2020) 258:118136 32726662 10.1016/j.lfs.2020.118136

[139] Mahdavi-Roshan M Salari A Ghorbani Z Ashouri A. The effects of regular consumption of green or black tea beverage on blood pressure in those with elevated blood pressure or hypertension: A systematic review and meta-analysis. Complement Ther Med. (2020) 51:102430 32507441 10.1016/j.ctim.2020.102430

[140] Biesinger S Michaels HA Quadros AS Qian Y Rabovsky AB Badger RS. A combination of isolated phytochemicals and botanical extracts lowers diastolic blood pressure in a randomized controlled trial of hypertensive subjects. Eur J Clin Nutr. (2016) 70:10–6. 26059745 10.1038/ejcn.2015.88

[141] Mi Y Liu X Tian H Liu H Li J Qi G. EGCG stimulates the recruitment of brite adipocytes, suppresses adipogenesis and counteracts TNF-α-triggered insulin resistance in adipocytes. Food Funct. (2018) 9:3374–86. 29868672 10.1039/c8fo00167g

[142] Ren Z Yang Z Lu Y Zhang R Yang H. Anti‑glycolipid disorder effect of epigallocatechin‑3‑gallate on high‑fat diet and STZ‑induced T2DM in mice. Mol Med Rep. (2020) 21:2475–83. 32236613 10.3892/mmr.2020.11041 PMC7185284

[143] Liang Y Wu F Wu D Zhu X Gao X Hu X. Fu Loose Tea Administration Ameliorates Obesity in High-Fat Diet-Fed C57BL/6J Mice: A Comparison with Fu Brick Tea and Orlistat. Foods. (2024) 13:206. 38254507 10.3390/foods13020206 PMC10815023

[144] Al-Rawaf HA Gabr SA Alghadir AH. Potential roles of circulating microRNAs in the healing of type 1 diabetic wounds treated with green tea extract: molecular and biochemical study. Heliyon. (2023) 9:e22020 38027999 10.1016/j.heliyon.2023.e22020 PMC10665742

[145] Xie L Tang Q Yao D Gu Q Zheng H Wang X. Effect of Decaffeinated Green Tea Polyphenols on Body Fat and Precocious Puberty in Obese Girls: A Randomized Controlled Trial. Front Endocrinol (Lausanne). (2021) 12:736724 34712203 10.3389/fendo.2021.736724 PMC8546255

[146] Garcia ML Pontes RB Nishi EE Ibuki FK Oliveira V Sawaya AC. The antioxidant effects of green tea reduces blood pressure and sympathoexcitation in an experimental model of hypertension. J Hypertens. (2017) 35:348–54. 28005704 10.1097/HJH.0000000000001149

[147] Sanae F Miyaichi Y Kizu H Hayashi H. Effects of catechins on vascular tone in rat thoracic aorta with endothelium. Life Sci. (2002) 71:2553–62. 12270760 10.1016/s0024-3205(02)02080-5

[148] Hinton T Jelinek HF Viengkhou V Johnston GA Matthews S. Effect of GABA-Fortified Oolong Tea on Reducing Stress in a University Student Cohort. Front Nutr. (2019) 6:27. 30972340 10.3389/fnut.2019.00027 PMC6443991

[149] Chen X Mu K Kitts DD. Characterization of phytochemical mixtures with inflammatory modulation potential from coffee leaves processed by green and black tea processing methods. Food Chem. (2019) 271:248–58. 30236674 10.1016/j.foodchem.2018.07.097

